# Brain transcriptome analysis of a CLN2 mouse model as a function of disease progression

**DOI:** 10.1186/s12974-021-02302-z

**Published:** 2021-11-08

**Authors:** Miriam S. Domowicz, Wen-Ching Chan, Patricia Claudio-Vázquez, Tatiana Gonzalez, Nancy B. Schwartz

**Affiliations:** 1 Department of Pediatrics, Biological Sciences Division, The University of Chicago Medical Center, 5841 S. Maryland Avenue, MC 5058, Chicago, IL 60637 USA; 2 Center for Research Informatics, Biological Sciences Division, The University of Chicago, Chicago, IL 60637 USA; 3 Department of Biochemistry and Molecular Biology, Biological Sciences Division, The University of Chicago, Chicago, IL 60637 USA

**Keywords:** Neuronal ceroid lipofuscinoses, Transcriptome, Lysosomal tripeptidyl peptidase 1, Pediatric neurodegeneration, Neuroinflammation, Choroid plexus

## Abstract

**Background:**

Neuronal ceroid lipofuscinoses, (NCLs or Batten disease) are a group of inherited, early onset, fatal neurodegenerative diseases associated with mutations in 13 genes. All forms of the disease are characterized by lysosomal accumulation of fluorescent storage material, as well as profound neurodegeneration, but the relationship of the various genes’ function to a single biological process is not obvious. In this study, we used a well-characterized mouse model of classical late infantile NCL (cLINCL) in which the tripeptidyl peptidase 1 (*Tpp1*) gene is disrupted by gene targeting, resulting in loss of detectable TPP1 activity and leading to progressive neurological phenotypes including ataxia, increased motor deficiency, and early death.

**Methods:**

In order to identify genes and pathways that may contribute to progression of the neurodegenerative process, we analyzed forebrain/midbrain and cerebellar transcriptional differences at 1, 2, 3 and 4 months of age in control and TPP1-deficient mice by global RNA-sequencing.

**Results:**

Progressive neurodegenerative inflammatory responses involving microglia, astrocytes and endothelial cells were observed, accompanied by activation of leukocyte extravasation signals and upregulation of nitric oxide production and reactive oxygen species. Several astrocytic (i.e., *Gfap, C4b, Osmr, Serpina3n*) and microglial (i.e., *Ctss, Itgb2, Itgax, Lyz2*) genes were identified as strong markers for assessing disease progression as they showed increased levels of expression in vivo over time. Furthermore, transient increased expression of choroid plexus genes was observed at 2 months in the lateral and fourth ventricle, highlighting an early role for the choroid plexus and cerebrospinal fluid in the disease pathology. Based on these gene expression changes, we concluded that neuroinflammation starts, for the most part, after 2 months in the *Tpp1*^*−/−*^ brain and that activation of microglia and astrocytes occur more rapidly in cerebellum than in the rest of the brain; confirming increased severity of inflammation in this region.

**Conclusions:**

These findings have led to a better understanding of cLINCL pathological onset and progression, which may aid in development of future therapeutic treatments for this disease.

**Supplementary Information:**

The online version contains supplementary material available at 10.1186/s12974-021-02302-z.

## Background

Neuronal ceroid lipofuscinoses (NCLs) are among the most common pediatric neurodegenerative diseases, causing progressive visual deterioration, intellectual and motor decline, epilepsy, ataxia, spasticity, and premature death. NCL incidence is estimated at 2–4 of 100,000 live births [1, 2] and usually is due to autosomal recessive mutations in genes implicated in the lysosomal pathway (13 genes to date) [3, 4]. Thus, they are classified as lysosomal storage disorders, are pathologically distinguished by unique intracellular fluorescent protein/lipid aggregates consisting of lipofuscin and the different forms are morphologically distinct at the electron microscopy level [3, 5].

Among NCL disorders, CLN2, also known as classical late infantile neuronal ceroid lipofuscinosis (cLINCL), is caused by mutations in the *TPP1* gene which encodes the lysosomal protease Tripeptidyl peptidase 1 (*TPP1*). The disorder is usually diagnosed between 2 and 4 years of age with onset of seizures and /or ataxia and early language delay. Severe neurological deterioration typical of NCLs follows, including blindness and progressive dementia, and most patients die between 7 and 15 years of age [6]. Diagnosis is usually confirmed by deficiency of TPP1 activity in leukocytes and/or identification of pathogenic mutations in *TPP1*. Interestingly, *TPP1* mutations which partially affect enzyme activity, have also been described in patients with spinocerebellar ataxia autosomal recessive 7 (SCAR7; OMID 609270). These patients, with 5% residual TPP1 activity in fibroblasts, have later disease onset than cLINCL patients (0.4% residual TPP1 activity), develop ataxia and cerebellar atrophy but not blindness or seizures [7].

Recent FDA-approved enzyme replacement therapy, currently the only available treatment, involves intracerebroventricular application of a recombinant human form of TPP1 (Brineura^R^, cerliponase alpha) and results in reduction of the motor-language score after 48 and 96 weeks of treatment [8]. This therapy could improve the quality of life for CLN2 patients if initiated earlier in the disease progression [9]. As well, an early clinical trial using adeno-associated virus (AAV)-mediated gene therapy showed that the rate of neurological decline was significantly slowed by the intracranial injection of AAV2 encoding hTPP1 in CLN2 patients [10]. Current clinical trials are exploring the use of an AAVrh.10 serotype which shows a broader distribution of viral transduction (NCT01161576, NCT01414985) [11].

TPP1 is a member of the S53 family of serine proteases that is synthetized as an inactive proenzyme and processed autocatalytically to the active form in the lysosome [12]. Some putative substrates have been characterized in vitro, including one of the main protein components of lipofuscin, the mitochondrial F_1_F_0_ ATP synthase, subunit C [3, 13, 14]. Interestingly, storage body accumulation does not correlate with neurodegeneration or astrocyte and microglia activation across the brain [15–17], and in fact, it has been suggested to be neuroprotective [16, 18]. Thus, neuroinflammation may be a better predictor of neuronal loss in several NCLs [18–20].

Some studies have analyzed the progression of neuroinflammation in different models of neurodegenerative disease (ND), and determined that ND leads to chronic neuroinflammation involving activated astrocyte and microglia; however, the mechanism of induction of neuroinflammation and the timelines of these processes differ between NDs [21–24]. Even though neuroinflammation has been recognized as an important characteristic at the pathological and cellular levels in NCLs [18, 25, 26], little is known about the onset of the neuroinflammatory process and the genes implicated. Furthermore, few molecular analysis timelines of transcriptional changes have been performed and only CLN1 has been analyzed as a function of disease development by microarray [27].

In order to perform a global brain and cerebellar RNA-seq analysis in a mouse model of CLN2 at the disease end-stage (4-month-old) [28], we used the well-established model of cLINCL generated in Lobel’s laboratory that targets the *Tpp1* mouse gene resulting in loss of detectable TPP1 activity and progressive neurological phenotypes including ataxia, motor deficiencies and blindness [29–33]. Homozygous affected animals have median survival of 19 weeks with initial motor deficiencies detected at 10 weeks using rocking rotating rod, open field motor and rearing activity and gait analysis [31, 33, 34]. At 4 months, our RNA-seq analysis revealed strong neurodegenerative inflammatory responses involving microglia and astrocytes, activation of leukocyte extravasation, dysregulation of neurotransmitter production, and upregulation of nitric oxide and reactive oxygen species; as well as downregulation of transcription factors involved in control of circadian rhythm [28]. In order to understand the nature and role of the neuroinflammatory response as well as to analyze regional and temporal vulnerabilities during disease progression, the present report extends our RNA-seq analysis to earlier time points including pre-symptomatic (1 and 2 months) and early symptomatic (3 months) stages of the disease in fore- and mid-brain (F/M) and cerebellum (Cb).

As reported, RNA-seq analysis of acutely purified different cell types potentially may give more specific transcriptional changes affecting cell populations [35]; however, this methodology has limitations since the cell purification steps per se may alter the transcriptional signature of certain cell types [36]. Alternatively, single-cell transcriptome analysis (scRNA-seq) may offer more reliable in vivo data, but it also has limitations such that the cells need to be physically separated before analysis and only a limited number of individual cells (3–5 thousand) can be evaluated [37]. In the present study, we performed transcriptome analysis of several brain areas to evaluate global transcriptional changes at different stages over disease evolution and assigned differentially expressed genes (DEG) on the basis of previously reported cell-specific expression and anatomical transcript distribution data available in the Allen atlas [38]. This approach has allowed us to define unique time-dependent progression of cell-specific gene expression and uncover early responses (i.e., in choroid plexus) that other methodological approaches would not have revealed. Also, our analysis establishes transcriptional changes in specific genes associated with neuroinflammation over time which will aid in identifying novel markers of disease evolution and in defining stage-specific therapeutic targets for treatment.

## Methods

### Animal procedures

All animal procedures were performed according to the University of Chicago Institutional Animal Care and Use Committee regulatory policies. Mice were housed under standard conditions, with access to food and water ad libitum*,* in a 12-h light and dark cycle. The mouse model for cLINCL, in which *Tpp1* is disrupted by gene targeting has been previously described [31] and is referred in this study as the *Tpp1*^*−/−*^ mutant. The mouse line was a generous gift from Drs. P. Lobel and D.E. Sleat. The line is being maintained by *Tpp1*^+/-^ heterozygous crosses. In all experiments *Tpp1*^−/−^ homozygous mice were compared to heterozygous (*Tpp1*^+/-^) or wild-type (*Tpp1*^+*/*+^) siblings of matched age and sex, as indicated.

Biological triplicates of total RNA were obtained from the cerebellum (Cb) and forebrain/midbrain (F/M) of one-, two- and three-month *Tpp1*^−/−^ and wild-type mice (*Tpp1*^+/-^ and *Tpp1*^+*/*+^, both genotypes have normal life span and principal component analysis of our RNA-seq studies do not segregate between them). TRIzol (Thermo Fisher Scientific) extraction was performed followed by a QIAGEN RNeasy Kit® clean-up procedure (included a DNAse-digestion step). RNA quality (RIN > 9.5) assessments, cDNA library preparation, and single-end sequencing with 50-nucleotide reads were performed by the University of Chicago Genomics Facility on Illumina platforms. RNA-sequencing files were transferred to the CRI’s Tarbell high-performance computing (HPC) cluster for analysis. Identical conditions used in the processing of 4-month RNA samples were published earlier and some data are included in this report for inclusiveness [28].

### Gene expression analysis

RNA-seq data analysis was performed by the University of Chicago CRI Bioinformatic Core as follows. The quality of raw sequencing data was assessed using FastQC v0.11.5 [39] and Illumina adapter/primer sequences were detected from sequencing reads. All RNA reads were first mapped to the mouse (mm10) reference genome using STAR v2.5.2b release with default parameters [40]. Picard v2.18.11 (http://broadinstitute.github.io/picard/) was used to collect mapping metrics. The resulting files from the previous alignment step in the RNA-seq analysis were taken individually as input to evaluate transcriptional expression using Rsubread::featureCounts v1.28.1 [41]. Data were inspected using normal distribution of GC content, principal component analysis, and normalized expression distribution. Afterwards, several methods of differential expression analysis (DEA); including edgeR v3.23.5 [42], DESeq2 v1.21.22 [43], and limma v3.45.5 [44] were employed to discover differentially expressed (DE) mRNAs between pair-wise groups based on expression estimation of individual mRNA genes (fold-change ≥ 1.5 and false discovery rate (FDR) < 0.1). Genes detected by all three DEA methods were collected to create a list of high-confidence DE genes (DEGs). To obtain the groups with similar expression trends based on identified DE genes, several in-house scripts were implemented using R (https://www.r-project.org/) and Perl (https://www.perl.org/) languages [45–47]. The identified DEGs were further used as input to functional analysis modules to identify enrichment of functional categories and regulatory networks using Gene Ontology (GO) terms and KEGG-enrichment analyses, as well as QIAGEN’s Ingenuity Pathway Analysis (IPA®) (www.qiagen.com/ingenuity). Pathways significantly enriched in the genes of interest were identified using clusterProfiler [48] (v3.6.0) at FDR-adjusted *p*-value < 0.10 (hypergeometric test). Gene Set Enrichment Analysis was performed using clusterProfiler [48] (v3.6.0), as well. It should be noted that due to the high level of stringency in the statistical analysis of RNA-seq for 2-, 3-, and 4- month-old tissue, the differences reported by this analysis may underestimate the total numbers of differentially expressed transcripts.

The cell-type (neuron, astrocyte, microglia, oligodendrocyte) specificities of the DEGs were determined based on transcriptome data from Zhang et. al. [49] as previously described [28]. DEGs considered to be enriched in the choroid plexus (ChPx) were assigned on the basis of published ChPx transcriptome analysis [50–53] and confirmed using the Allen Mouse Brain Atlas (https://mouse.brain-map.org/).

### mRNA in situ hybridization

Control and *Tpp1* mutant 2-month brains were fixed in 4% paraformaldehyde and processed for non-radioactive in situ hybridization as described previously [54, 55]. To prepare the digoxigenin (DIG)-labeled RNA probes used for ISH, cDNA fragments from *Lipopolysaccharide binding protein* (*Lbp*;) and *Klotho* (*Kl*) genes were generated by PCR and inserted into pCRII dual-promoter vector plasmids (Invitrogen) Lbp probe: nt 360–1229 of NM 008489.2 and Kl probe: nt 3505–4495 of NM 013823.2. Sequencing of the cloned gene fragments was performed with an ABI PRISM 377XL sequencer (Perkin Elmer) by the University of Chicago Cancer Center DNA sequencing facility. Riboprobes incorporating DIG-labeled nucleotides were synthesized from linearized PCR templates with SP6 or T7 RNA polymerase (Roche). Probed mRNAs were detected after hybridization with an alkaline-phosphatase-conjugated anti-DIG antibody (Roche). Alkaline phosphatase activity was detected (blue color) using BCIP and NBT substrates (Roche). Images were obtained using an Axiovert 200 microscope (Zeiss).

### Immunohistochemistry

Control and *Tpp1* mutant 2-month and 4-month brains were fixed in 4% paraformaldehyde and processed for in situ hybridization as described, but instead of being hybridized, sections were blocked with 10% normal lamb serum for 2 h and incubated with a 1:500 dilution of ATP synthase subunit C antibody (Abcam ab181243) overnight at 4 °C. A FITC-conjugated anti-mouse IgG was used as secondary antibody and slices were counterstained with DAPI. A virtual library of the images was obtained using a CRi Pannoramic MIDI 20X Whole Slide Scanner (Perkin Elmer) and final pictures were selected using the CaseViewer Software. Quantification of the immunostaining was performed using FIJI software and statistical significances were evaluated by ANOVA analysis followed by Tukey test using Vassar University statistical computation site (http://vassarstats.net/).

### Quantitative PCR

RNA purified as described above was reverse-transcribed with the High Capacity Reverse Transcription Kit (AppliedBiosystems) and RT-qPCRs were performed using the Sso Advanced TM Universal SYBR Green system (Bio-Rad Laboratories) in a CFX-96 real time instrument (Bio-Rad Laboratories). For each gene, forward and reverse primers were designed using Primer-BLAST (NCBI) and later tested in RT-qPCR; only primer pairs with 90–105% efficiency were utilized. Primers for *Kl, Clic6* and *Prlr* were purchase from Origene. Relative normalized expression values were determined using the ΔΔCt methods for the indicated target genes relative to *Ppia* (*Peptidylprolyl isomerase A*) in the wild-type mouse sample. The data are expressed as means ± standard deviations. Determinations were performed in triplicate and experiments were repeated with independent RNA samples at least 3 times with consistent results. Statistical significances were evaluated by the paired-samples Student’s *t*-test using the CSF-96 BioRad software package. Values of *p* < 0.05 for the null hypothesis were considered significant. Primers used were as follows: *Ppia* (Fwd: 5′-GCTGGACCAAACACAAAC-3′, Rev: 5′-CCACAATGTTCATGCCTTC-3′), *Aqp1*(Fwd: 5′-CTTGCCATTGGCTTGTCTGTGG-3′, Rev: 5′-CCAGTGGTTTGAGAAGTTGCGG-3′), *Ttr* (Fwd: 5′-GAGTAGAACTGGACACCAAATCG-3′, Rev: 5′-CTGCGATGGTGTAGTGGCGATG-3′). Ppia was used for single normalization since our RNA-seq data do not indicate changes of this gene when compared at any specific age.

## Results

### Global gene changes at different ages

Using the cLINCL mouse model in which *Tpp1* was disrupted by gene targeting, we analyzed the progression of the disease in forebrain/midbrain (F/M) and cerebellum (Cb) by performing.

RNA-seq from RNA obtained at 1-, 2-, 3-, and 4-month *Tpp1*^*−/*−^ (T) animals and compared them to matched age controls (N). Each age was processed as previously reported [28]. At 1 and 2 months, affected animals were asymptomatic, as the earliest indications of abnormal motor phenotypes are only evident after 10 weeks of age [31, 33, 34]. By 3 months, disease phenotypes progress rapidly with the development of several motor defects and pathological hallmarks [30, 31, 33], followed by death after 4 months. Note that the 4-month results have been published elsewhere, but selected data are included in this report for completeness [28].

Table 1 summarizes the number of DEGs at different ages and shows a fold-change ≥ 1.5 and FDR < 0.1, indicating the percentage of genes mapped and the total number of genes analyzed in each data set. Raw and processed RNA-seq data are available from the National Center for Biotechnology Information Gene Expression Omnibus under accession number: GSE173665 and the differentially expressed gene list at each age can be found in Additional file 1.

**Table 1:** Summary of differentially expresses genes resulting from RNA-seq comparisons of cerebellum (Cb) and fore- and mid-brain (F/M) regions between *Tpp1*^*−/−*^ (T) and control animals (C) at different ages

Month	% of reads mapped	Mouse gene analyzed	Brain region	DEG T vs C	DEG down T vs C	DEG up T vs C
1*	88.7	14,681	F/M	2 (126)	2 (84)	0 (42)
			Cb	2 (255)	2 (65)	0 (190)
2	87.2	14,081	F/M	139	25	114
			Cb	339	292	47
3	88.1	14,575	F/M	89	11	78
			Cb	154	31	123
4	88.3	14,923	F/M	510	171	339
			Cb	1550	553	1071

*At 1 month few DEGs genes were detected using FDR-corrected *p*-values; raw *p*-values were considered instead in the threshold (*p* < 0.1) only at 1-month-old (number in parenthesis)

**Fig. 1:**
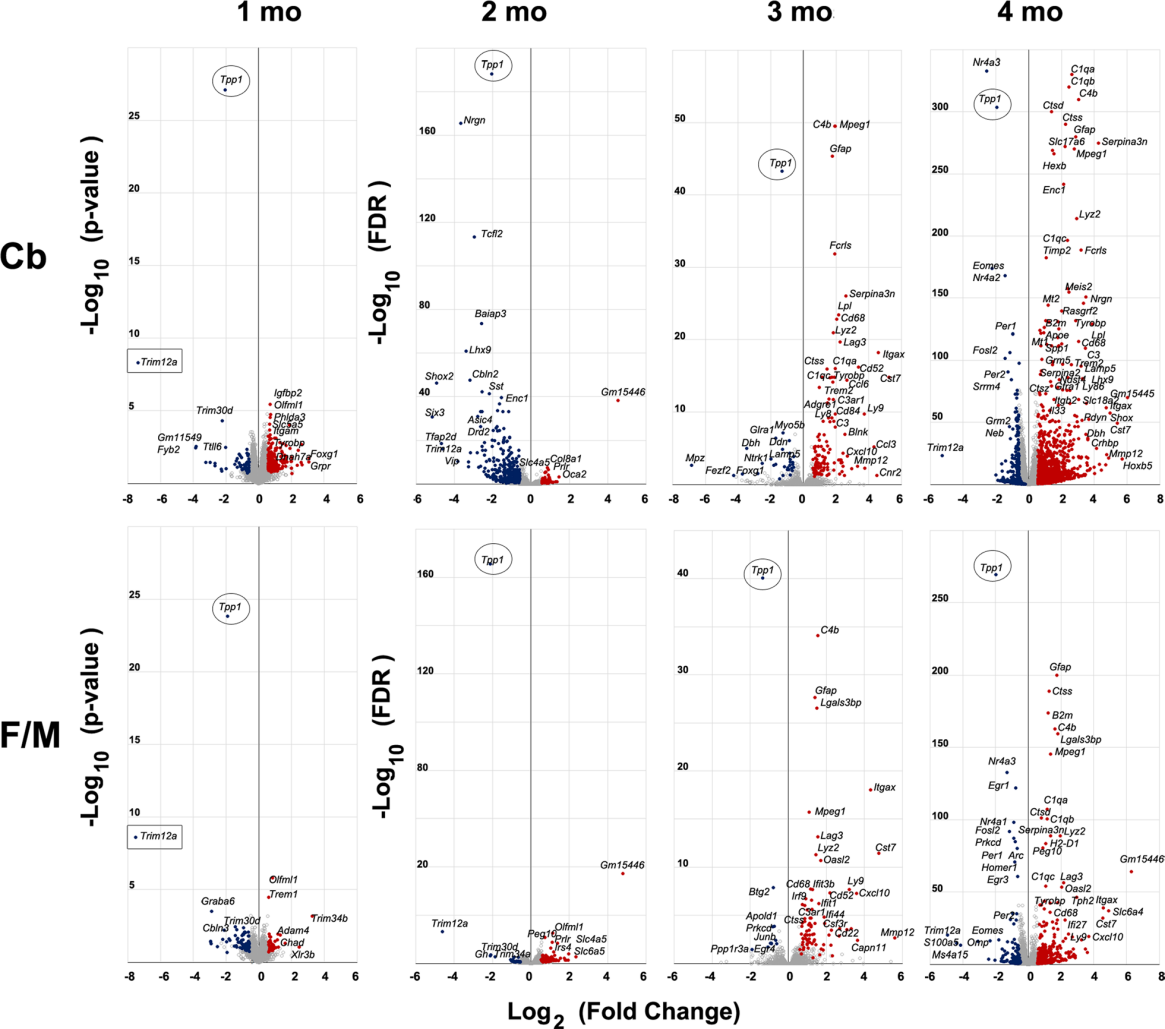
Volcano plot. Representation of differentially expressed genes (FDR ≤ 0.1 and fold-change ≥ 1.5) at 2-, 3- and 4-month-old cerebellum (Cb) and fore- and mid-brain (F/M) regions between *Tpp1*^*−/−*^ and control animals. Selected downregulated genes (blue dots) and upregulated genes (red dots) are designated. The rest of the genes are represented with gray open circles. Tpp1 levels are indicated with a circle. For one-month-old animals the −log_10_ of the *p* value was represented instead. For this data set, Tpp1 and Trim 12a (rectangle) were found to be significantly downregulated

**Fig. 2:**
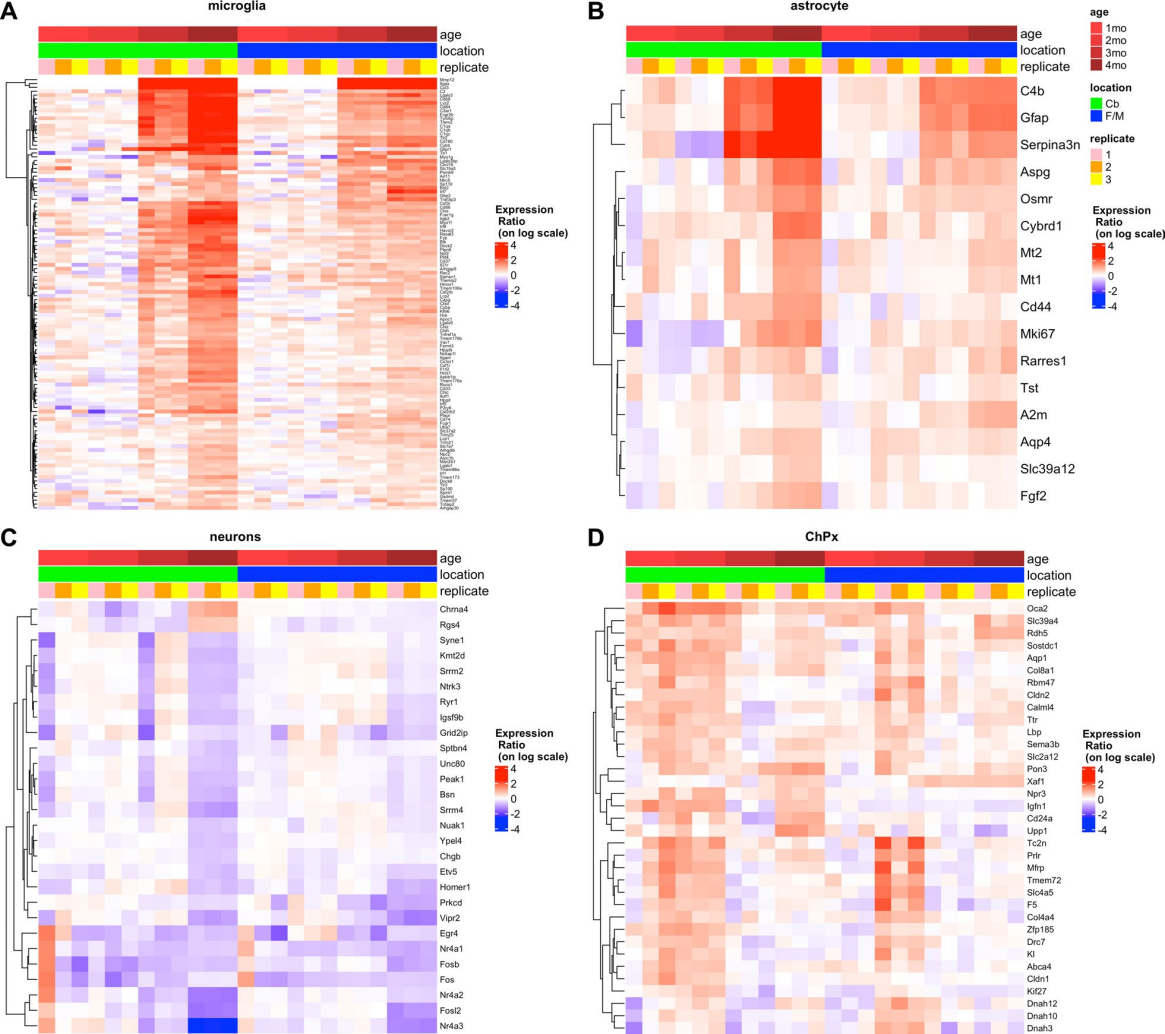
Heatmap with clustering of cell-type specific DEG in *Tpp1*^−/−^ brain regions as a function of time. Distribution of cell-type specific genes that are commonly enriched in DEG from Cb-T vs. Cb-N (green) and F/M-T vs. F/M-N (blue) comparisons at 1, 2, 3, 4 months (different degrees of reds). Gene expression ratios (FPKM T/ average FPKM N) are represented in logarithmic scale. Cell-type **A** microglia, **B** astrocytes and **C** neurons) specificity of DEG was established from data imputed from Zhang et al. [49] based on genes that were exclusively expressed in each cell type and had FPKM expression values at least five times higher than in the rest of the other cell types. Choroid plexus genes (**D**) where classified by expression values from Turner et al., 2014 [53]

At 1 month few DEGs genes were detected using FDR-corrected *p*-values; thus, in order to recover more DEGs, raw *p*-values were considered instead in the threshold (*p* < 0.1), but since these time point data lack the rigor imposed to the rest of the study, we only included the one-month set in Fig. 2, and the data were excluded from the rest of the analysis. The number of DEGs in the cerebellar samples is consistently greater than in the rest of the brain (*p* < 0.0001 using significance of the difference of two independent proportions), suggesting that the cerebellum is more affected in this disease than the rest of the brain; this was subsequently ascertained by canonical pathway analysis (see below). Volcano plots highlight upregulated (red) and downregulated (blue) genes in the *Tpp1*^*−/−*^ brains at the different ages compared to control (Fig. 1). As expected, Tpp1 is always highly downregulated in the mutant cohort at all ages. The increased number of upregulated genes at 3- and 4-month brains reflects the developing neuroinflammatory response toward end-stages of the disease (see below). As a way to visualize this finding, we identified the cell-type enrichment profile of the DEGs found at each age, based on the cell-type transcriptomes previously reported [49] (Additional file 2). We further analyzed the unassigned DEG expression patterns in the Allen Mouse Brain Atlas, and found that at 1 and 2 months of age several of the DEGs were expressed strongly or exclusively in the ChPx. Thus, using transcriptome analysis from human and mouse ChPx [50, 53], we compared which of the DEGs were also among the 3000 genes highly expressed in ChPx and displayed those in Additional file 2 (green). The results indicate an involvement of the ChPx associated with the third ventricle (cerebellar region) during the asymptomatic periods of the disease (1 and 2 months) and in the lateral ventricles (F/M region) at 2 months of age. These observations were also confirmed by enrichment analysis (SGEA) which demonstrated an enrichment score (ES) for Cb of 0.76 (FDR q-value < 0.0001) and an ES for F/M of 0.68 (FDR q-value < 0.0001) for choroid plexus genes (Lein Choroid Plexus Markers, M1719).

### Microglia-enriched mRNAs

In order to analyze the evolution of the expression of selected genes from each cell type during disease progression, their gene expression ratios (FPKM T/ average FPKM N) were displayed as heatmaps with clustering as a function of time (Fig. 2). These analyses allowed us to identify gene signatures associated with the different cell types and determine how they changed with disease progression. Additional file 3 offers an alternative view of the consolidated data with better definition of the fold changes. As mentioned, neuroinflammatory markers start to be upregulated at 3 months and are preferentially expressed in microglia and astrocytes (Fig. 2A, B). The most upregulated transcripts in microglia are *Mmp12*, *Itgax* and *Ccl3*, which show significant changes at 3 and 4 months, but not at 1 or 2 months. Many other microglial genes were also upregulated, and for the most part the levels of change were more accentuated in Cb than in F/M. Several microglial genes are not significantly upregulated at 3 months, but do already show increased expression, indicating their involvement in microglia activation. Among the highlighted genes, the microglia receptor Trem2 and its adaptor protein DAP12 (encoded by *Tyrobp*) initiate a signal transduction pathway that promotes microglia phagocytosis, chemotaxis and proliferation [56–58]. We also observed transcriptional upregulation starting at 3 months in the affected animals of many components of the complement pathway including *C1qa*, *C1qb*, *C1qc* and *C3* and its receptor *C3ar1*, among others. During normal development, synaptic pruning is performed by microglia through the complement pathway [59, 60] and this pathway has also been implicated in clearance of amyloid plaques, neurofibrillary tangles and synapses in Alzheimer disease (AD) [61, 62]. Still, it remains to be determined if increases of complement pathway components in the *Tpp1*^*−/−*^ brains are affecting the clearance of lipofuscin storage material, neuronal spines and/or apoptotic cells.

Upregulation of Toll-like receptors (*Tlr1* and *Tlr2*) were also observed in the *Tpp1*^*−/−*^ brains. These receptors usually respond to disease-associated molecular patterns (DAMPs) leading to a proinflammatory response and activation of the NLRP3 inflammasome pathway. While *Tlr2* is upregulated at 3 months, other components of the inflammasome pathway such as *Nlrp3*, *Nlrp1b* and *Gmdsd* are increased at the end stage of the disease (4-month-old) [28] when chronic inflammation is evident.

A sharp increase in the transcriptional levels of *Matrix metalloproteinase-12* (*Mmp12*), in *Tpp1*^*−/−*^ animals between 2 and 3 months was observed. Mmp12 has been reported to be involved in increasing blood–brain barrier permeability and recruitment of macrophages in inflammatory conditions and plays a role in models of experimental autoimmune encephalomyelitis (EAE) by enhancing the anti-inflammatory response [63, 64]. Mmp12 is a zinc-binding protease expressed by microglia and infiltrated macrophages in disease-caused neuroinflammation and aging [65–67]; thus, this transcript may be one of the better indicators of inflammatory initiation.

CD11c or Integrin αX (encoded by the *Itgax* gene) is also upregulated in *Tpp1*^*−/−*^ animals at post-symptomatic stages. It is also controlled by the TREM activation pathway [58] and has been recognized as a marker of “prime” microglia (pre-activation stage) in aging and ND [68, 69].

*Ccl3*, together with other members of the macrophage inflammatory protein-1 family (i.e., *Ccl4* and *Ccl9*), are also strongly upregulated in *Tpp1*^*−/−*^ brains at 3 and 4 months of age. *Ccl3* has been reported to induce expression of *Ccr5*, its potential receptor, in brain–blood barrier endothelial cells and increase T cell trans-endothelial migration from the blood to the brain in AD [70]. It is possible that similar mechanisms take place in the *Tpp1*^*−/−*^ cerebellum since we observed increased transcription of *Ccr5* in this area at 4 months.

Increased expression of *Cd68* which encodes for a lysosomal macrophagic protein indicative of phagocytic activity [71], was also detected at 3–4 months in *Tpp1*^*−/−*^ mutants.

### Astrocyte-enriched mRNAs

Among of the highly upregulated genes in astrocytes are those previously identified as reactive associated genes [72, 73]. C4b, Serpina3n and GFAP are the most highly upregulated astrocytic genes in *Tpp1*^*−/−*^ F/M and  Cb (Fig. 2B). Interestingly, C4b and Serpina3n are also upregulated in cortical astrocytes with aging and are attributed to A1 astrocyte activation [74] that may trigger some synapse loss with normal aging and disease [75, 76]. Clearly, their expression curve which closely resembles that of GFAP, makes these two genes important markers of disease progression (see “Discussion”).

Transcriptional analysis of astrocytes in models of bacterial infection (lipopolysaccharide injection) and ischemic tissue damage have allowed for the determination of astrocytic gene markers upregulated in reactive astrocyte (PAN-reactive), and genes that define the specific reactive phenotypes of A1- and A2-astrocytes [72, 73]. When we analyzed changes in these genes during the progression of CLN2 disease, an upregulation of PAN- and A1-reactive genes was observed at 3 months; however, fewer A2-reactive genes were upregulated, but do appear to increase later at 4 months (Additional file 4). Since A2-reactive astrocytes have been proposed to be neuroprotective and important for mitigating neuroinflammation, this observation may indicate a failure to mount an effective anti-inflammatory response during the early symptomatic period of CLN2 disease.

We also observed upregulation of metallothionein I and II (*Mt1* and *Mt2*) zinc-binding proteins expressed by astrocytes, which play roles in metal homeostasis and free radical scavenging [77], and have been postulated to promote neural regeneration and survival following injury [78, 79].

### Neuronal-enriched mRNAs

With few exceptions, loss of transcripts involved in neuronal function correlates with increased neuroinflammation. Several members of the Fos and NUR families are downregulated as CLN2 disease progresses (Fig. 2C). These are immediate early genes involved in transcriptional regulation that are regulated by neuronal activity. *Fos*, *Fosb* and *Fosl2* encode for leucine zipper proteins that when dimerized with Jun, form AP-1 transcription factor complexes. In particular, c-Fos (encoded by *Fos*) and ∆FosB (a splicing form of *Fosb*) are better studied in brain as they are activated in response to synaptic stimulation, thereby mediating synaptic, structural plasticity and neuronal circuit remodeling in the striatum and nucleus accumbens [80, 81]. Interestingly, *Fos* was downregulated in both brain areas analyzed as early as 2 months.

Three members of the Nur transcription factor subfamily *Nr4a1* (Nur77), *Nr4a2* (Nurr1) and *Nr4a3* (Nor-1) are downregulated as CLN2 disease progresses; in particular, *Nr4a1* is downregulated as early as 2 months in the F/M region (Fig. 2C). These are orphan members of the nuclear receptor superfamily and can form heterodimers with each other or with the RXR retinoid receptor family. They are expressed in the motivation/reward circuit and the HPA axis where they play an integrative regulatory signaling role during stress and addiction [82]. Furthermore, levels of *Nr4a2* are downregulated in AD patients and in several AD mouse models [83–85].

**Fig. 3:**
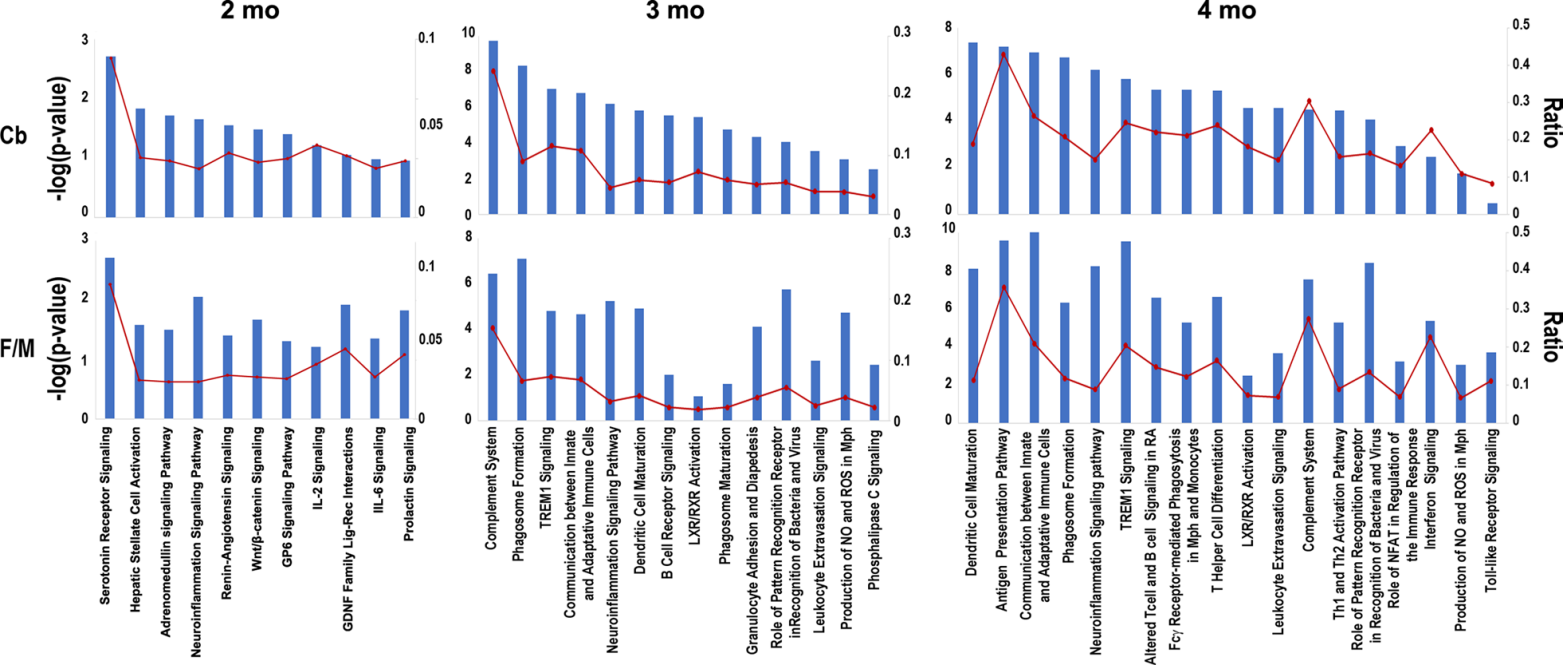
Ingenuity pathways analysis (IPA). DEG found in *Tpp1*^*−/−*^ vs control mouse cerebellum (Cb) and fore- and mid-brain (F/M) regions were used in the analysis. Selected canonical pathways were identified using IPA gene ontology algorithms for F/M and Cb areas scored as − log(*p*-value) from Fisher’s exact test, set here to a threshold of 1.25. The ratio (red dots connected by a line) indicates the ratio of genes from the data set that map to the pathway divided by the total number of genes that map to the same pathway. For a complete list of canonical pathways and genes involved, see Additional file 5

Other members of the neuronal immediate early genes include activity-dependent transcription factors from the ERG family [86], which are also affected as disease progresses, especially in the F/M regions (see Erg4 in Fig. 3C). Also, cytomatrix proteins of the presynaptic active zone (i.e., *Bassoon* and *Piccolo*) are downregulated after 3 months of age, indicating structural presynaptic compromise late in disease progression.

### Choroid plexus genes

One of the most surprising findings of this comprehensive RNA-seq analysis during the pre-symptomatic periods (1–2 months) is the upregulation of many genes expressed almost exclusively in the ChPx (Fig. 2D). In the F/M region which includes the lateral and third ventricle, a significant transcriptional increase in 29 genes expressed in the ChPx was observed. In the Cb region, ChPx genes from the fourth ventricle were also increased significantly by 2 months; however, increases in some of these genes were also detected, albeit not significantly, as early as 1 month of age. Thus, these subtle changes in the fourth ventricle ChPx are the first observed transcriptional changes in diseased brains. The genes include: structural extracellular matrix genes (i.e., *Col4a4*, *Col8a1*); secreted proteins (i.e., *Coagulation factor 5* (F5), *Klotho* (Kl), *Sclerostin domain-containing protein 1* (*Sostdc1*), *Lipopolysaccharide binding protein* (*Lbp*), *Paraoxonase 3* (*Pon3*), *Transthyretin* (*Ttr*)); transporters (i.e., *Sodium bicarbonate cotransporter* (*Slc4a5*), *Melanocyte-specific transporter protein* (*Oca2*), A*quaporin 1* (*Aqp1*), *Chloride intracellular channel 6* (*Clic6*), *Glucose transporter type 12* (*Slc2a12*)); tight junction components (i.e., *Claudin 2* (*Cldn2*)); receptors (i.e., *Prolactin receptor* (*Prlr*), *Folate receptor alpha* (*Folr1*)); integral component of secretory epithelium membrane (i.e., *Tmem72*); enzymes (*Retinol dehydrogenase 5* (*Rdh5*).

As well, the cilia component (*Dynein regulatory complex Subunit 7* (*Drc7*) was upregulated in cerebellum. Other upregulated cilia components were identified by EnrichGo in the F/M region (i.e., *Adenylate kinase 7* (*Ak7*), several dynein heavy chains (including *Dnah10, 11, 12, 3, 6* and *7b*) and *Sperm flagellar protein 2* (*Spef2*)). These components are involved in cilia motility, characteristic of the ependymal cells lining the ventricles [87]. In contrast, ChPx cells contain non-motile (9 + 0 configuration) cilia whose function is still not clearly understood [88, 89].

### Pathway analysis

**Fig. 4:**
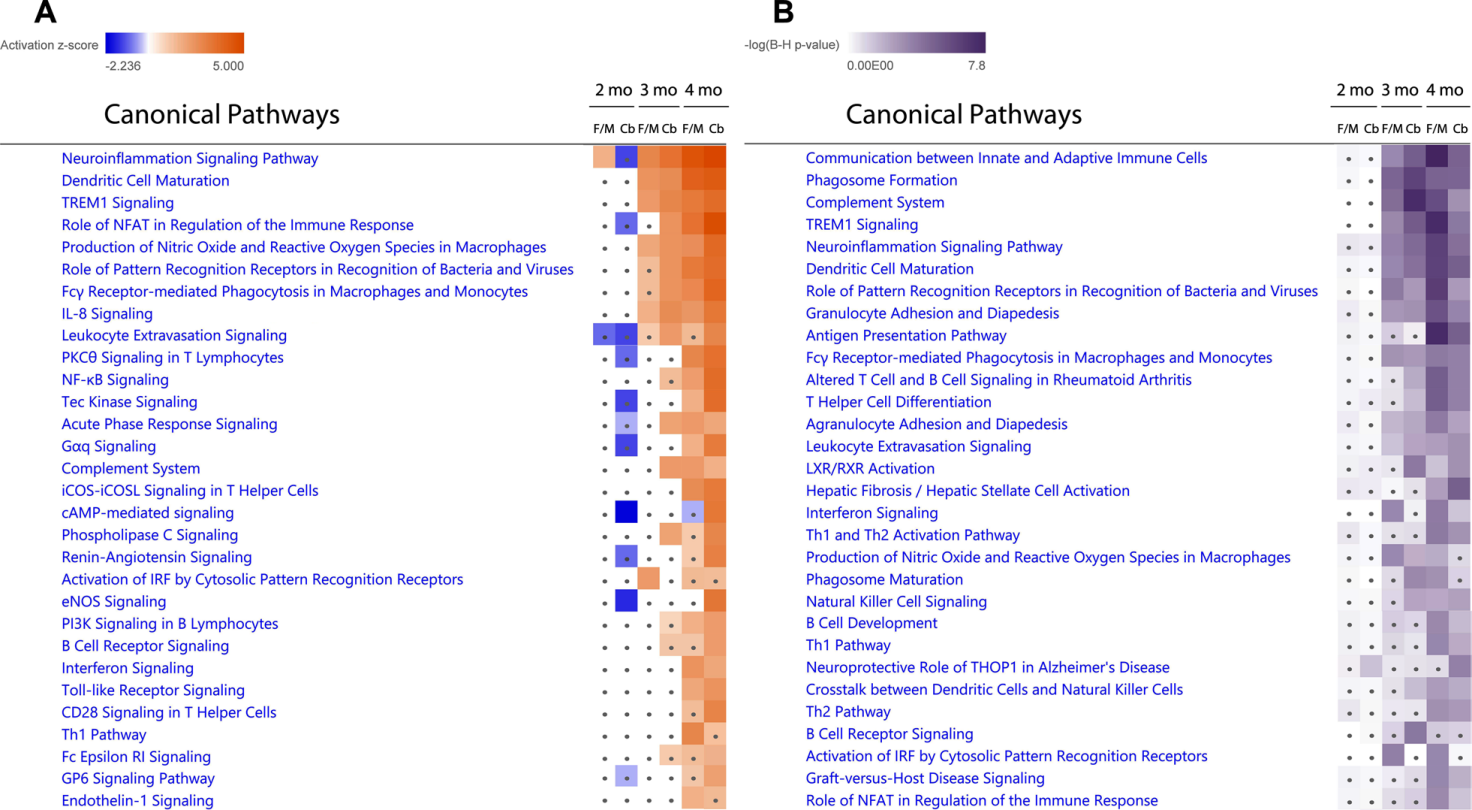
Comparison analysis of canonical pathways across ages using IPA. DEGs found in *Tpp1*^*−/−*^ vs control mouse cerebellum (Cb) and fore- and mid-brain (F/M) regions at 2–4 months were used in the analysis. Thirty top z-scores (**A**) and – log of Benjamini–Hochberg corrected *p*-value (**B**) are represented as a heatmap. Orange and blue colored *z*-scores represent activation and suppression, respectively. Dots indicate activation scores below 1.64 for A and corrected *p* > 0.05 for B

Ingenuity pathway analysis (IPA) identified functional canonical pathways that are linked to DEGs in control vs *Tpp1*^*−/−*^ brain regions at different ages (2–4 months). Analysis at 1 month is not presented since there were only a few significant DEGs found. A selection of the biologically relevant pathways affected by the CLN2 disease model are depicted in Fig. 3 and the full list of pathways, their activation scores and genes implicated are provided in Additional file 5. Only 2 pathways are consistently identified at all ages, i.e., neuroinflammation signaling and LXR/RXR activation. As disease progresses, the number of genes associated with the neuroinflammation signaling pathway increases, perhaps denoting the establishment of a chronic inflammation profile. Also, unlike at 2 months, a closely related number of pathways largely implicated in the immune response are involved in the symptomatic period of the disease (3–4 months) (Additional file 6) including: TREM signaling, phagosome formation, leukocyte extravasation signaling and production of NO and ROS signaling. In order to confirm these age pair-wise observations, a comparison analysis of the canonical pathways across all ages was performed on the IPA platform. Results indicate that the neuroinflammation signaling pathway, dendritic cell maturation, TREM1 signaling, role of NFAT in immune response regulation, and production of NO and ROS in macrophages are the most highly activated pathways (positive z-score) at 3 and 4 months in both brain areas analyzed (Fig. 4A), while stronger statistical changes were associated with communication between innate and adaptive immune cells, phagosome formation, complement system, Trem1 signaling and neuroinflammation signaling pathways (Fig. 4B). It is also important to notice that the z-scores increased with age and that they are higher in Cb than in F/M for all of these prominent pathways confirming our aforementioned assertions that Cb is more affected by the lack of Tpp1 and that neuroinflammation increases with age.

Analysis of upstream regulators identified the proinflammatory cytokine interferon gamma (IFNG) as one of the possible regulators of the inflammatory response progression at 3–4 months in both F/M and Cb (Cb 4 months: *p*-value: 1.6e^−26^, Activation *z*-score: 7.1; F/M 4 months: *p*-value: 9.9e^−33^, Activation z-score: 6.3; Cb 3 months: *p*-value: 1.1e^−17^, Activation *z*-score: 4.0; F/M 3 months: *p*-value: 3.8e^−18^, Activation *z*-score: 3.5) leading to TLR2 activation and NFkB complex formation indicated by their mechanistic network (Additional file 7). At 2 months, the estrogen receptor 1 (Esr1) is identified as an upstream regulator (F/M *p*-value: 7.7e^−10^, Activation *z*-score: 2.; Cb *p*-value: 2.7e^−13^, Activation *z*-score: 3.1), linking for the most part ChPx components (Additional file 8). Upstream regulator comparisons across all ages and brain areas was performed and top 30 *z*-score and *p* values are represented in Additional file 9. Please note that only the genes marked with arrows were detected to also change their transcriptional levels at either 3 of 4 months of age. This analysis confirms IFNG and IFNAR (interferon α/β receptor) as the most significant upstream regulators of disease progression with positive *z*-scores that increase with age.

IPA analysis of the neurological disease networks showed that while inflammation of the nervous system is the statistically highest networks associations at 3 and 4 months in *Tpp1*^*−/−*^ Cb and F/M regions, at 2 months, abnormal morphology of the nervous system and seizures were the most prominent associations in F/M and Cb, respectively (Additional file 10).

### Analysis of choroid plexus alterations at 2 months

**Fig. 5:**
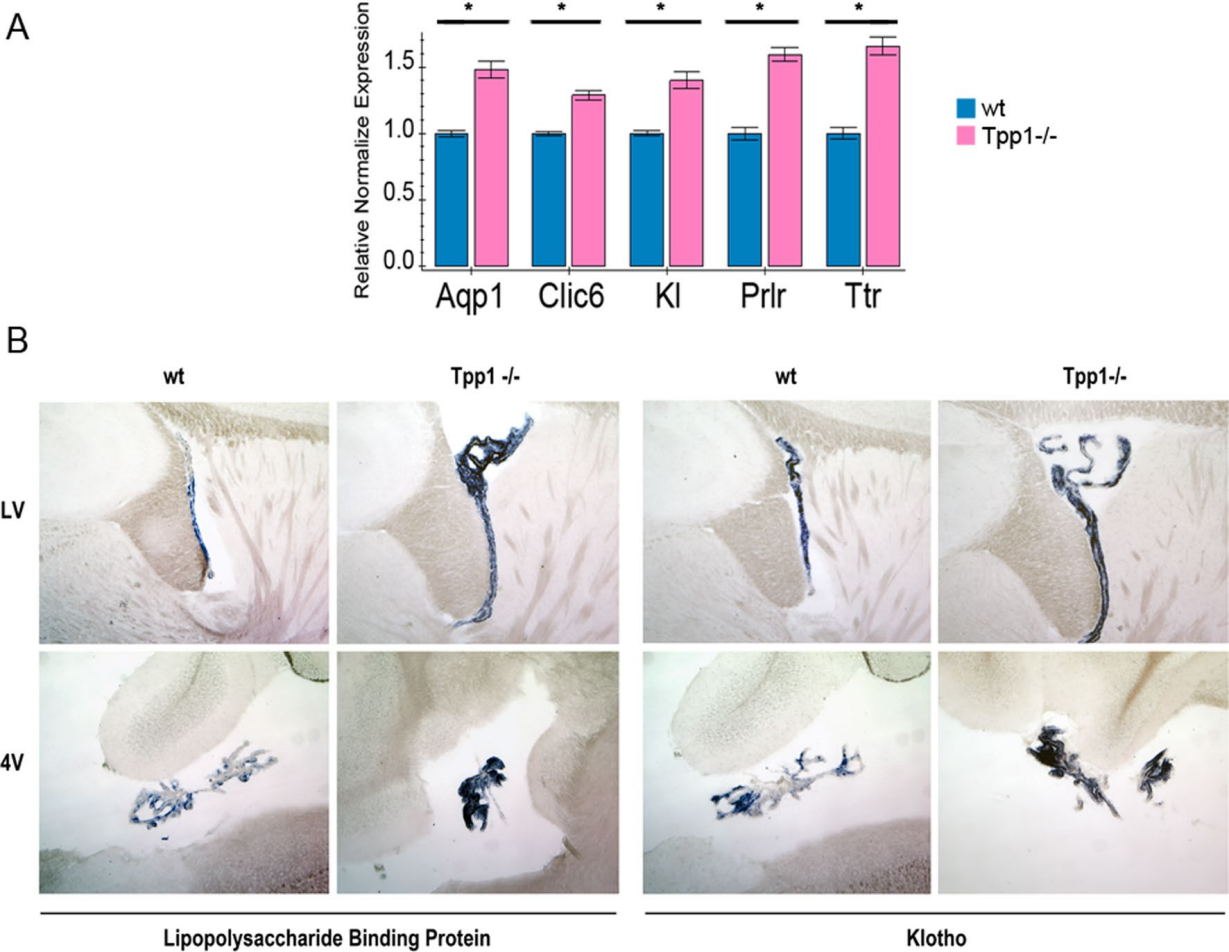
Increase in choroid plexus gene expression in *Tpp1*^−/−^ brains. **A** Relative transcript expression of 5 genes upregulated in *Tpp1*^*−/−*^ choroid plexus were quantified by qPCR in independent F/M RNA extractions, performed in triplicate. Relative normalized expression values were determined from the ΔΔCt for the indicated target genes relative to Ppia (peptidylprolyl isomerase A) expression in control brains (wt). *p < 0.001; **B** Expression pattern of *Lipopolysaccharide binding protein* and *Klotho* (blue staining) in the lateral and 4th ventricle of *Tpp1*^*−/−*^ and control choroid plexus detected by mRNA in situ hybridization

As mentioned, about 29 ChPx expressed genes were found to be upregulated in 2-month-old brains of *Tpp1*^*−/−*^ animals. We further confirmed some of these gene expression differences by qPCR and mRNA in situ hybridization. Transcript levels of *Aqp1, Clic6, Kl, Prlr* and *Ttr* were upregulated at 2 months by qPCR in the F/M area (Fig. 5A). As well, Kl and Lbp mRNAs showed increased levels in the epithelial layer of the ChPx located in the lateral and fourth ventricles of *Tpp1*^*−/−*^ brains by in situ hybridization (Fig. 5B). It is important to note that although 4-month Tpp1^−/−^ brains always showed enlarged lateral ventricles, probably due to thinning of the cortex, this is rarely observed in 2-month-old brains.

We also explored the possibility of increased cell division in 2-month ChPx from *Tpp1*^*−/−*^ brains by BrdU incorporation and Ki67 staining. As previously reported, cell division is seldom observed in adult ChPx brains [90, 91], and this was also confirmed for ChPx in *Tpp1*^*−/−*^ animals (data not shown). Thus, it was not possible to perform quantitative analysis of the proliferative cells, but allowed us to conclude that increased epithelial proliferation does not account for the transcriptional upregulations observed in ChPx of *Tpp1*^*−/−*^ brains.

**Fig. 6:**
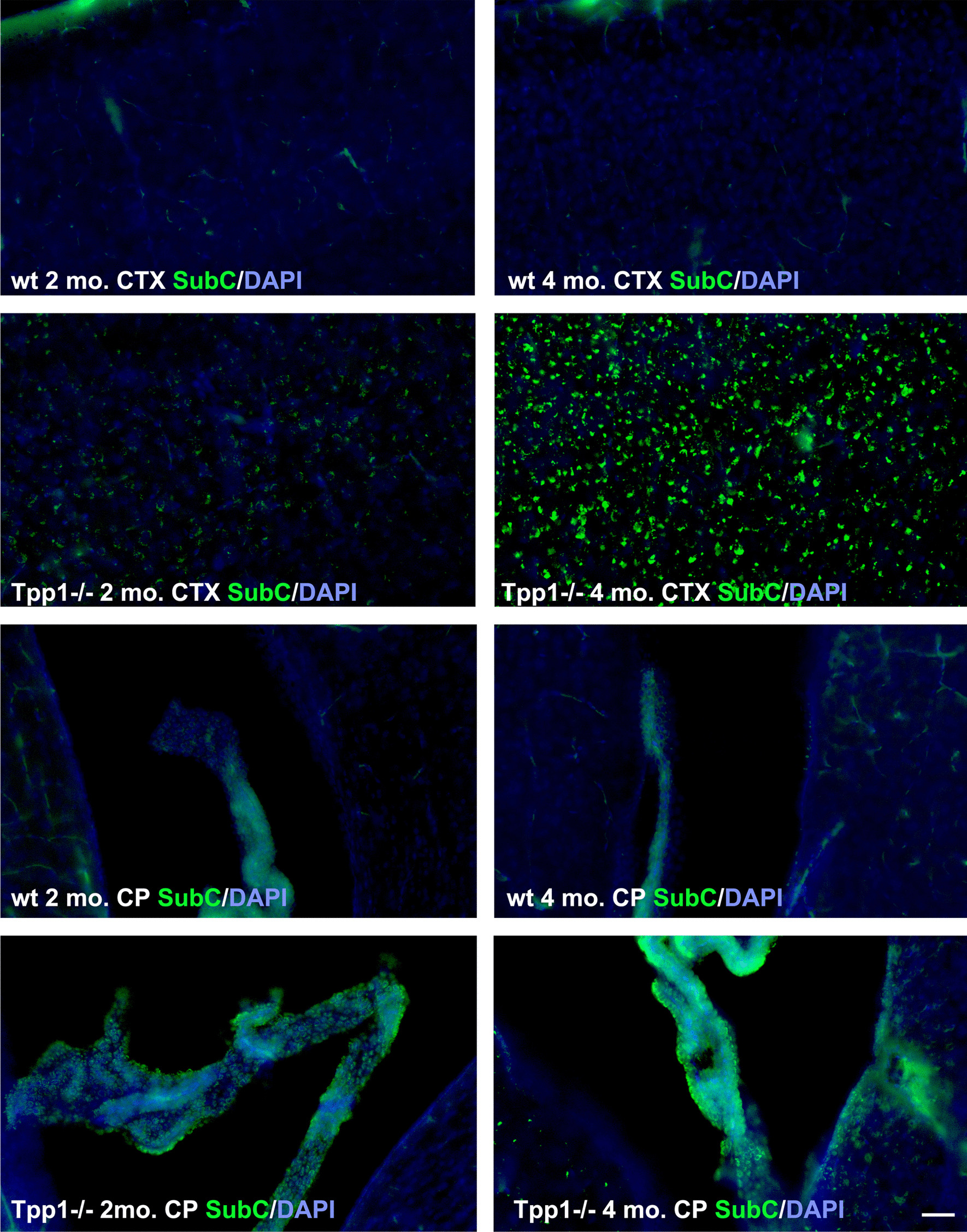
Mitochondrial *ATP synthase subunit C* (SubC) accumulation over time. Immunohistochemistry of SubC in *Tpp1*^*−/−*^ and control (wt) cortex (CTX) and choroid plexus (CP) at 2 and 4 months of age stained with antibody ab181243 (green). Nuclei were stained with DAPI. Quantification of the SubC expression was performed using Fiji software in three independent brains and it is displayed in Additional file 11

Lastly, we established whether lack of *Tpp1*^*−/−*^ per se could be affecting the ChPx epithelium by staining with anti-mitochondrial *F*_1_*F*_0_ ATP synthase, subunit C antibody (SubC) in order to follow lipofuscin accumulation. Levels of SubC were significantly increased in the cortex of 2-month-old *Tpp1*^*−/−*^ brains compared to wt, and expression substantially increased by 4 months as determine by the number of cells and the area stained (Fig. 6, Additional file 11). Similar to cortex, ChPx epithelium expressed detectable SubC levels at 2 months and significantly higher levels at 4 months, suggesting that the lack of Tpp1 could be affecting lysosomal functions in ChPx as early as 2 months of age (Fig. 6, Additional file 11).

## Discussion

Like most NCLs, the CLN2 form of this disorder is a complex and fast-developing neurodegenerative disease for which the affected mechanistic pathways remain largely unknown. Taking a systems biology approach, we evaluated the transcriptional changes using RNA-seq in two major brain areas, F/M and Cb, at pre- and post-symptomatic stages of the disease in order to establish a more comprehensive understanding of disease progression, as well as to better define regional and cell-type specific involvement.

In vitro, TPP1 cleaves multiple peptide hormones, i.e., angiotensin II [92], glucagon [93], substance P [92], cholecystokinin and neuromedin [94]) as well as amyloid-β and the mitochondrial ATP synthase subunit C [13]. Interestingly, none of the transcripts for these substrates were altered at any age in *Tpp1*^*−/−*^ brains, perhaps because the initial steps of the disease are at the level of lysosomal dysfunction. As well, several lysosomal genes, identified by GSEA analysis, were found to be increased only at 3 and 4 months of age in both Cb and F/M areas, including two NCL genes, cathepsin D and Granulin. However, because many of these genes have been shown to be upregulated in activated microglia and astrocytes, it is difficult to ascertain if these transcriptional changes can be attributed to lysosomal dysfunction.

Proteomic analysis in brains of CLN1-, CLN2- and CLN3-animal models detected a considerably smaller number of changes in protein expression at terminal stages of these genetic disorders than the magnitude of DEGs detected by our study, perhaps due in part to the limited sensitivity of proteomic analyses [95]. Nonetheless, several proteins altered in our transcriptomic analysis (LYZ2, TGM1, GPNMB, C4B, SERPINA3N, GFAP and C1QC) were confirmed to be increased in the proteomics analysis of CLN2 and were related to microglial and astrocytic function [32]. Interestingly, proteomic profiling of spinal cord in CLN1 disease (Ppt1^−/−^ model) identified canonical pathways consistent with the ones identified in this study for the *Tpp1*^*−/−*^ brains [96]; as well, notable regional differences were found in this study.

Our transcriptome analysis indicates that waves of events occur over developmental time. There is initial activation of ChPx function at 2 months, followed by an acute increase in inflammatory responses that include: activation of microglia and astrocytes, increased production of nitric oxide and reactive oxide species and activation of leukocyte extravasation. Between 2 and 4 months transcriptional downregulation of several immediate early genes known to be regulated by neuronal activity are observed culminating in downregulation of cytomatrix proteins of the presynaptic active zone as well as circadian rhythm transcription factors in 4-month brains. Furthermore, comparing cerebellum and F/M responses during disease progression highlight both conserved and divergent mechanisms, considering the cytoarchitectural and biochemical difference in these tissues. An accelerated response of astrocytic and microglial genes was observed in cerebellum marked by larger quantitative changes in gene expression that may be contributing to the neuronal damage at terminal stages. It should be noticed that some neuronal immediate early genes (i.e. fos and Nr4a1) start to be downregulated prior to the establishment of neuroinflammation. Future experiments should extend this analysis to other strongly affected areas such as thalamus, ponds and spinal cord.

With regard to the immune inflammation response, the first microglial component found to be consistently downregulated in the *Tpp1*^*−/−*^ model at 1 month in F/M and Cb is *Trim12a*. Tripartite motif containing proteins (TRIM proteins) belong to the E3 ubiquitin ligase family and play a crucial role in innate immunity [97]. *Trime12a* is a member of an expanded paralogous cluster of Trim5-like proteins in mice which are known to be antiviral host genes that act against a range of retroviruses; however, their functions in regulating neuroinflammation have not been studied thus far. By 2 months, *Trim12a*, *Trim30d* and *Trim34a* are significantly downregulated in both areas analyzed. However, it is not until the third month that a clear immunological inflammatory response is established involving the complement system, phagosome formation and maturation, TREM2 signaling, production of NO and ROS by macrophages and leukocyte extravasation signaling. These responses are further enhanced by 4 months of age in the mutant line [28]. Furthermore, evidence of stage 1 (TREM2 independent) and stage 2 (TREM2 dependent) disease-associated microglia (DAM) transcriptional signatures as described for Alzheimer disease models [98], start to be evident as early as 3 months in *Tpp1*^*−/−*^ brains.

Comparable to other neurodegenerative diseases, in this model of NCL the initial danger-associated molecular pattern (DAMP) signals [99] (likely lipofuscin) seems to be sufficiently accumulated by 2 months to trigger the early events that mount the immune response which is demonstrable at 3 months, driven by microglia and astrocyte activation and accompanied by declining neuronal function, that then continues to be accentuated as the disease progresses. In Cln3^−/−^ and Ppt1^−/−^ mouse lines, it has been proposed that impaired astrocyte and microglia function, as well as neuron–glia communication are in part responsible for disease progression [100, 101]. In this*Tpp1*^*−/−*^ model the classical transcriptional responses from activated microglia and astrocytes observed in other types of brain injuries were confirmed [24, 35, 73], while failure to mount an effective anti-inflammatory response during disease progression may ultimately contribute to the neuronal damage by A1 astrocytes [72]. This observation may explain why the activation of microglia and astrocytes may be more accurate predictors of neuronal loss than the accumulation of lipofuscin [102, 103].

The major predicted upstream regulators in this study were IFNG and IFNAR, but neither molecule was mapped in the 2-, 3- or 4- month RNA-seq data. Thus, this insight suggests a new avenue for future mechanistic research in NCLs in defining therapeutic targets.

The second unique aspect of the inflammatory response in this model is the accelerated timeline of the microglia and astrocytic response that is also reflected in an increased regional response between cerebellum and the rest of the brain. To put this in perspective, during normal aging *Gfap* levels increase three times in mouse cerebellum from 4 months to 2 years of age [74], in Alzheimer mouse models levels increase 5 times in 9-month-olds [104] and in the Tpp1, model *Gfap* increases 7 times in 4 months, emphasizing the accelerated pathology. Among the astrocytic genes, *Serpina3n*, which belongs to the serine protease inhibitor (serpin) superfamily, illustrates the largest expression changes in the cerebellum with increases up to 20 times. *Serpina3n* is designated an inflammatory marker expressed in brain astrocytes [73] and is best known in the immune system for regulating the inflammatory response by inhibiting the activity of leukocyte cathepsin G released at the site of inflammation [105]. Although, it has been consistently found to be increased in several neurodegenerative diseases (i.e., Alzheimer disease, Parkinson disease) and in brain injury, its mode of action is not clearly understood [106–109]. Recently, single-nucleus transcriptome analysis identified a previously discovered *Serpina3n* + *C4b* + reactive oligodendrocyte population in AD brains [110]. Confirmation that increased expression of *Serpina3n* and *C4b* in *Tpp1*^*−/−*^ brains is associated with reactive astrocytes requires further examination.

Perhaps the most unique finding of our analysis relates to the early times points (1–2 months) when upregulation of ChPx genes may highlight a key function for this tissue in the initiation of the disease. ChPx is a profusely vascularized structure located in the ventricular system that is not only responsible for the production of a large fraction of the CSF, but also contributes many homeostatic functions to the brain, including: establishment of the CSF-blood barrier; synthesis and secretion of signaling factors, growth factors and hormone receptors; and formation of the neuroimmune gateway allowing entry of adaptive immune cells to the CSF thereby regulating CNS immunosurveillance [111, 112]. Interestingly, increases in Cldn2 and Aqp1 have been reported to indicate a decrease of leakiness of the ChPx epithelium [112], while increased expression of Kl and Ttr may reflect a neuroprotective response by the ChPx [113, 114]. It is not clear why there is early, but transient, upregulation of certain genes expressed in the choroid plexus epithelium. A few possibilities are proposed: (i) since the ChPx has been shown to be an alternative entry site for cells of the immune system to the CNS during immune surveillance and neuroinflammation [115], this surveillance function may detect the initial lipofuscin accumulation. In fact, we have demonstrated that detectable levels of subunit C in the ChPx epithelium at 2 months coincide with the initial ChPx response (Fig. 6). Thus, it will be important to determine if increased extravasation of immune cells accompany this activation. (ii) Alternatively, increased expression of genes associated with the secretory function of the ChPx could reflect the initiation of the ventricular enlargement in the *Tpp1*^*−/−*^ brain even though actual ventricular enlargement is evident only after 3 months of age. Since the major ciliated cell types in brain are ependymal cells lining the ventricles and the ChPx epithelium itself, it is also interesting that upregulation of ChPx functions coincide with increased expression levels of structural cilia components, suggesting a possible activation of cilia movement.

While knowledge is limited of how a neuroinflammation response is initially mounted during neurodegeneration, our findings suggest the ChPx is a possible site of origin for the immune surveillance response and/or an initial line of defense by offering increased neuroprotection, at least in NCLs. Thus, it would be important to determine if other neurodegenerative diseases also elicit transient ChPx changes, or if this observation is unique to this model. Thus far, analysis of choroid plexus transcriptome in post-mortem AD, MS, FTD and HD indicates deleterious as well as adaptive changes in the ChPx [116–118], and ChPx dysfunction that impairs β-amyloid clearance has been documented in animal models of Alzheimer disease [119].

## Conclusions

Elucidating the biological foundation and pathological mechanisms of neurodegenerative diseases are key to development of targeted therapies to slow or prevent the onset of disease sequelae in CLNs. Our findings establish a timeline of events initiated in pre-symptomatic *Tpp1*^*−/−*^ brains that highlight a role for the choroid plexus during early disease stages and subsequent development of an accelerated inflammatory response involving astrocytes and microglia activation. De-escalating the inflammatory response may prove to be an important therapeutic path to explore, as it may limit the neuronal damage accompanying this response. Most importantly, this study provides a framework outlining potential therapeutic windows, and opens novel areas for further investigation in the NCLs, as well as other neurodegenerative diseases with common underlying pathological mechanisms.

## Supplementary Information

**Additional file 1.** List of expressed genes identified by RNA-seq in forebrain /midbrain (F/M) and cerebellum (Cb) in Tpp1^−/−^ mouse brains (T) versus control (N) brains at 1-, 2- and 3- and 4-month of age.

**Additional file 2.** Identification of cell-type specific DEG in wt and Tpp1^−/−^ brain region.

**Additional file 3.** Ratio of genes expressed in microglia, astrocytes, neurons, and choroid plexus over time.

**Additional file 4.** Relative differential expression of activated astrocyte signature genes (FPKM in Tpp1^−/−^ relative to control) at different ages.

**Additional file 5.** Ingenuity Pathway Analysis (IPA) of genes differentially expressed in forebrain /midbrain (F/M) and cerebellum (Cb) in Tpp1^−/−^ mouse brains versus control brains at 1-, 2- and 3- and 4-month of age. Each age data set is a different excel sheet.

**Additional file 6.** Overlapping Canonical pathways at 2-, 3- and 4 months of age in forebrain/midbrain (F/M) and cerebellum (Cb) identified by IPA analysis.

**Additional file 7.** Upstream analysis of Interferon gamma mechanistic network at 3 and 4 months of age in forebrain/midbrain (F/M) and Cerebellum (Cb) identified by IPA analysis.

**Additional file 8.** Upstream regulation of estrogen receptor 1 at 2 months of age in forebrain/midbrain (F/M) and Cerebellum (Cb) identified by IPA analysis.

**Additional file 9.** Comparison analysis of upstream regulators across ages using IPA.

**Additional file 10.** Neurological Disease Network identified by IPA analysis affected in the Tpp1^−/−^ brain. DEG involved in each category are included. Each age data set is a different excel sheet.

**Additional file 11.** Quantification of the number of SubC positive cells and area stained per 0.1 mm^2^ was performed in cortex of three Tpp1^−/−^ and control brains at 2 and 4 month-old age.

## Data Availability

Raw and processed RNA-seq data are available from the National Center for Biotechnology Information Gene Expression Omnibus under accession number GSE173665.

## Abbreviations

NCLs: Neuronal ceroid lipofuscinoses
*Tpp1*, *TPP1*: *Tripeptidyl peptidase 1* (Tpp1, mouse; TPP1, human)
DEG: Differentially expressed genes
IPA: Ingenuity pathway analysis
F/M: Forebrain/midbrain
Cb: Cerebellum
ChPx: Choroid plexus
RNA-seq: RNA sequencing
FDR: False discovery rate
L2fc: Log twofold change
