# Supplementary figures and images for: Brain transcriptome analysis of a CLN2 mouse model as a function of disease progression
Source: J Neuroinflammation. 2021 Nov 8;18:262. doi: 10.1186/s12974-021-02302-z (PMC8576919; doi:10.1186/s12974-021-02302-z)

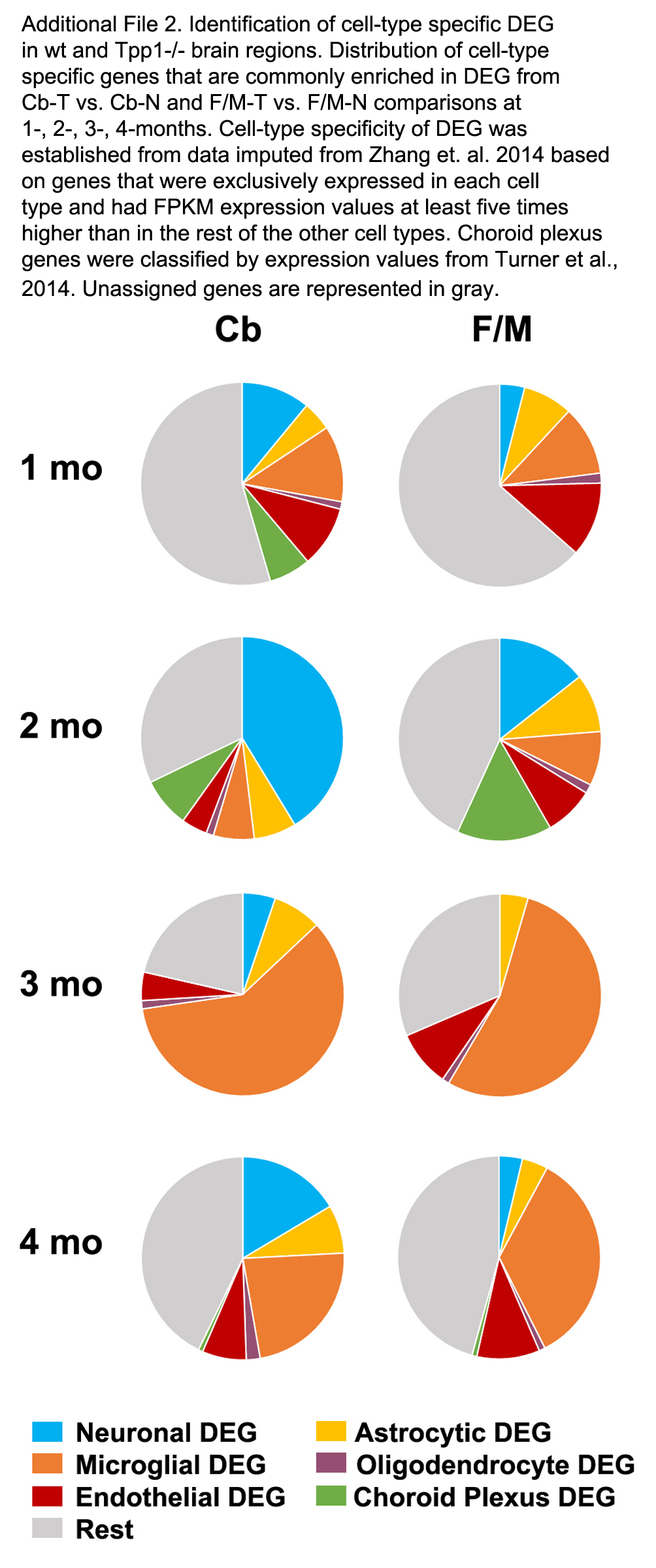

Supplement: Supplementary file 2 — Additional file 2. Identification of cell-type specific DEG in wt and Tpp1−/− brain region. [file 12974_2021_2302_MOESM2_ESM.tif]

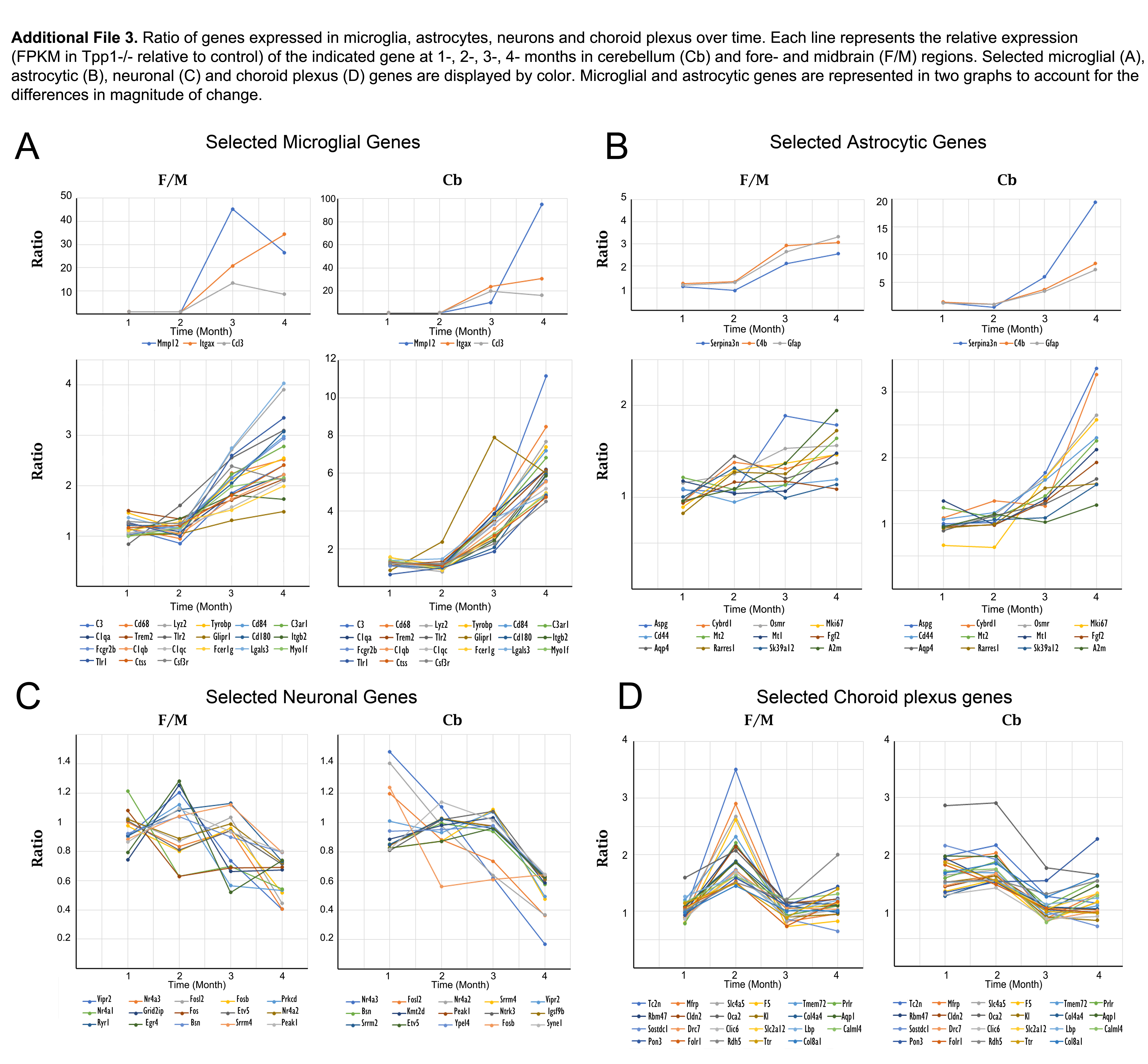

Supplement: Supplementary file 3 — Additional file 3. Ratio of genes expressed in microglia, astrocytes, neurons, and choroid plexus over time. [file 12974_2021_2302_MOESM3_ESM.tif]

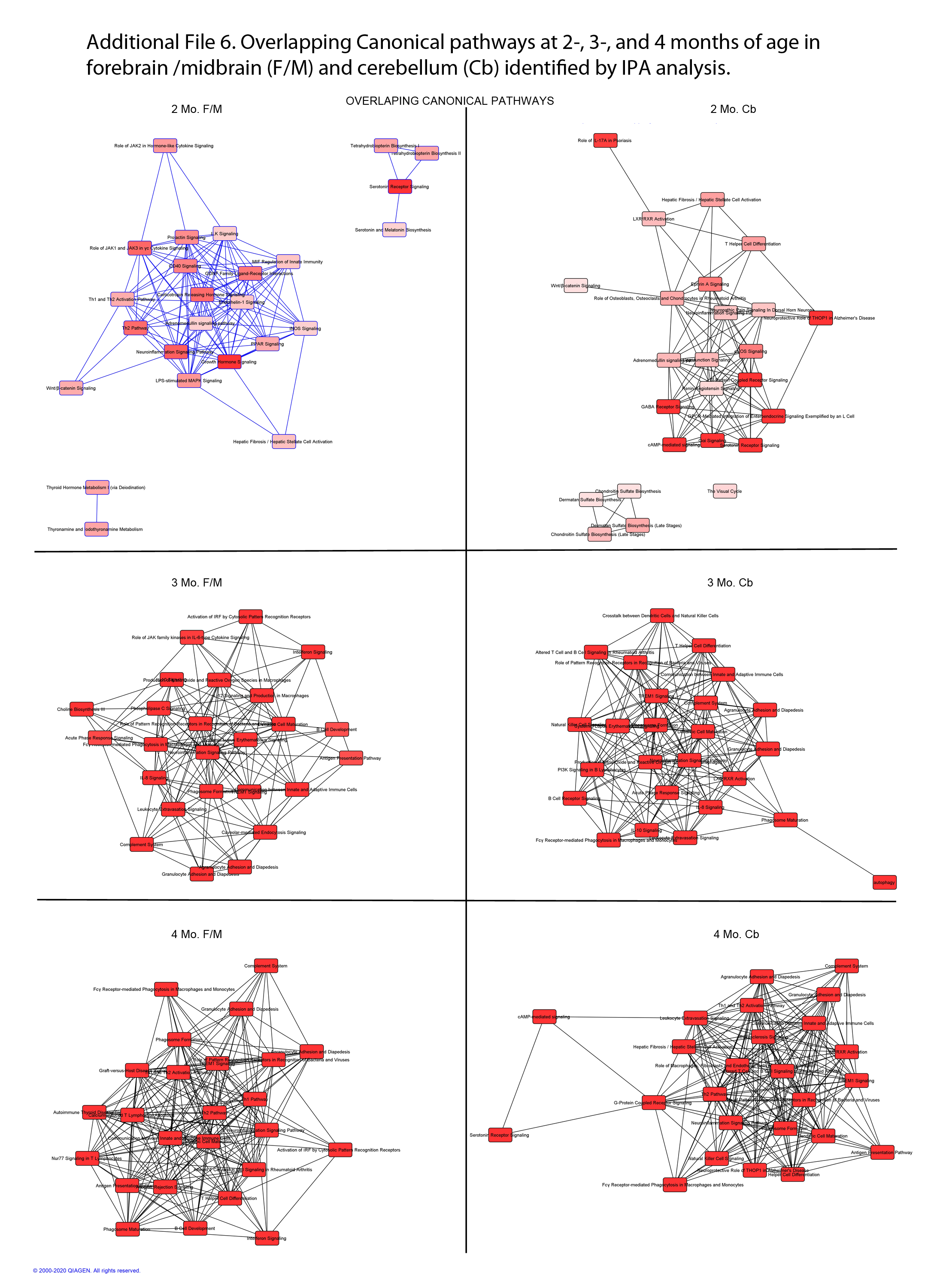

Supplement: Supplementary file 6 — Additional file 6. Overlapping Canonical pathways at 2-, 3- and 4 months of age in forebrain/midbrain (F/M) and cerebellum (Cb) identified by IPA analysis. [file 12974_2021_2302_MOESM6_ESM.tif]

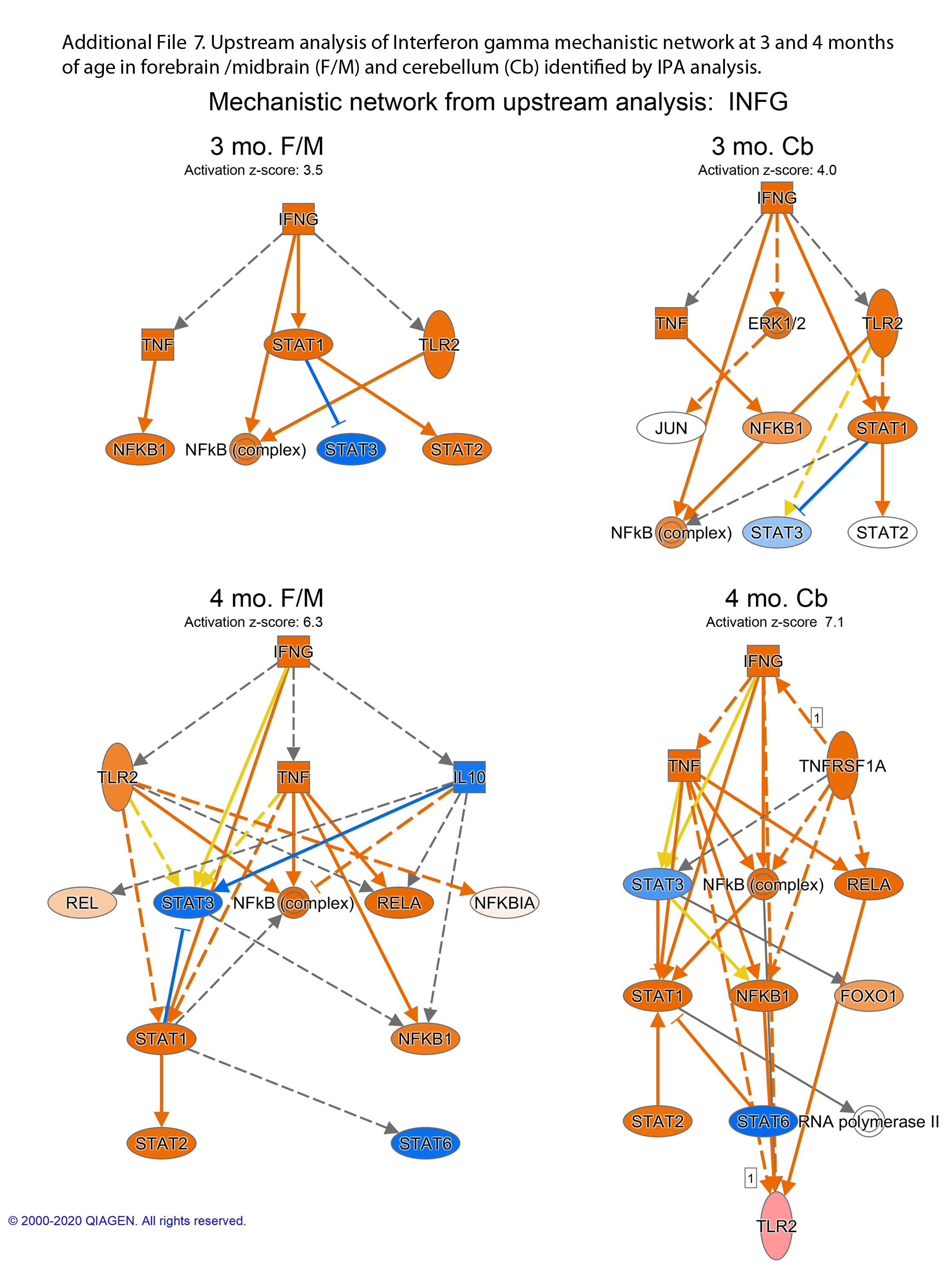

Supplement: Supplementary file 7 — Additional file 7. Upstream analysis of Interferon gamma mechanistic network at 3 and 4 months of age in forebrain/midbrain (F/M) and Cerebellum (Cb) identified by IPA analysis. [file 12974_2021_2302_MOESM7_ESM.tif]

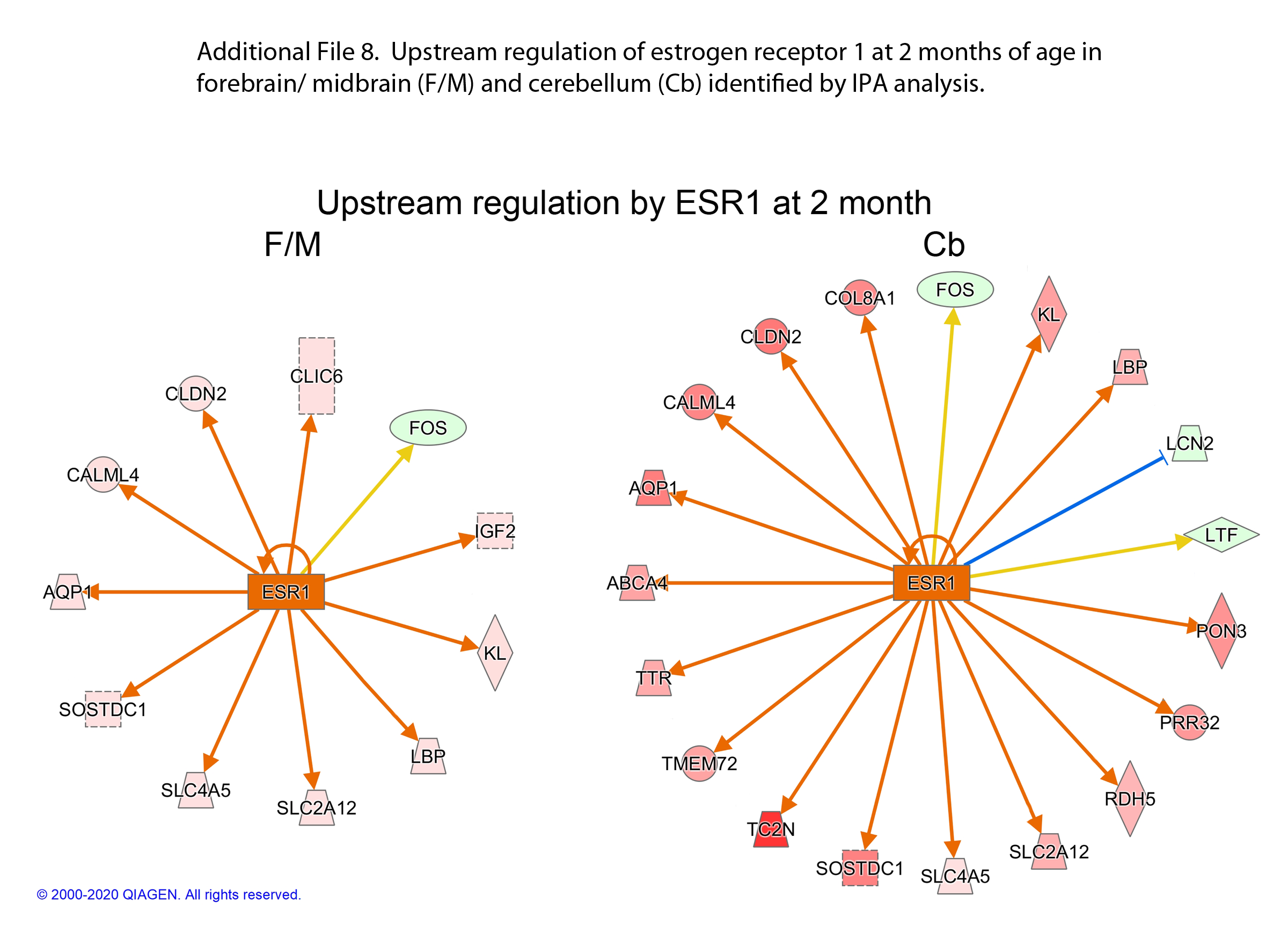

Supplement: Supplementary file 8 — Additional file 8. Upstream regulation of estrogen receptor 1 at 2 months of age in forebrain/midbrain (F/M) and Cerebellum (Cb) identified by IPA analysis. [file 12974_2021_2302_MOESM8_ESM.tif]

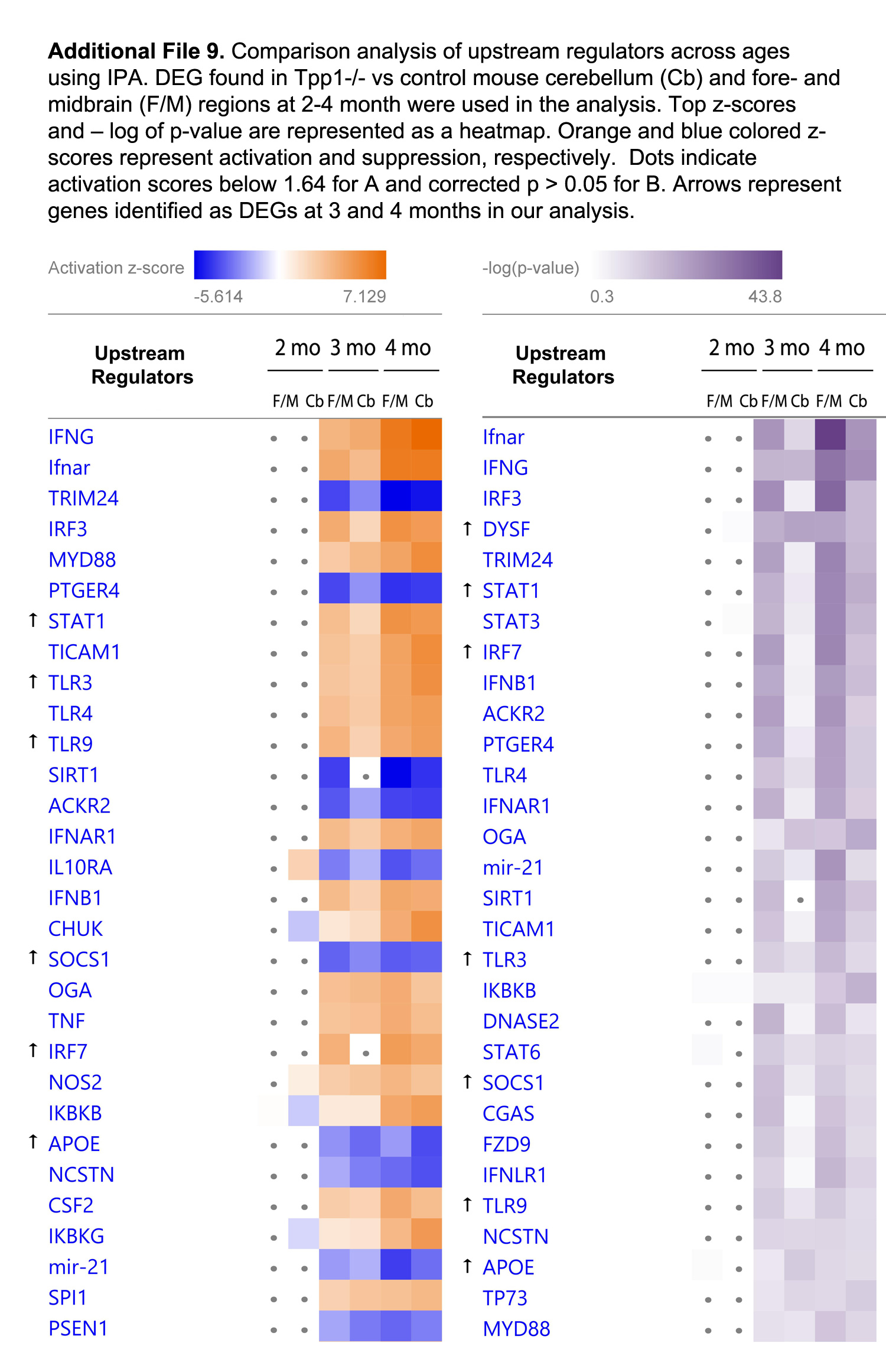

Supplement: Supplementary file 9 — Additional file 9. Comparison analysis of upstream regulators across ages using IPA. [file 12974_2021_2302_MOESM9_ESM.tif]

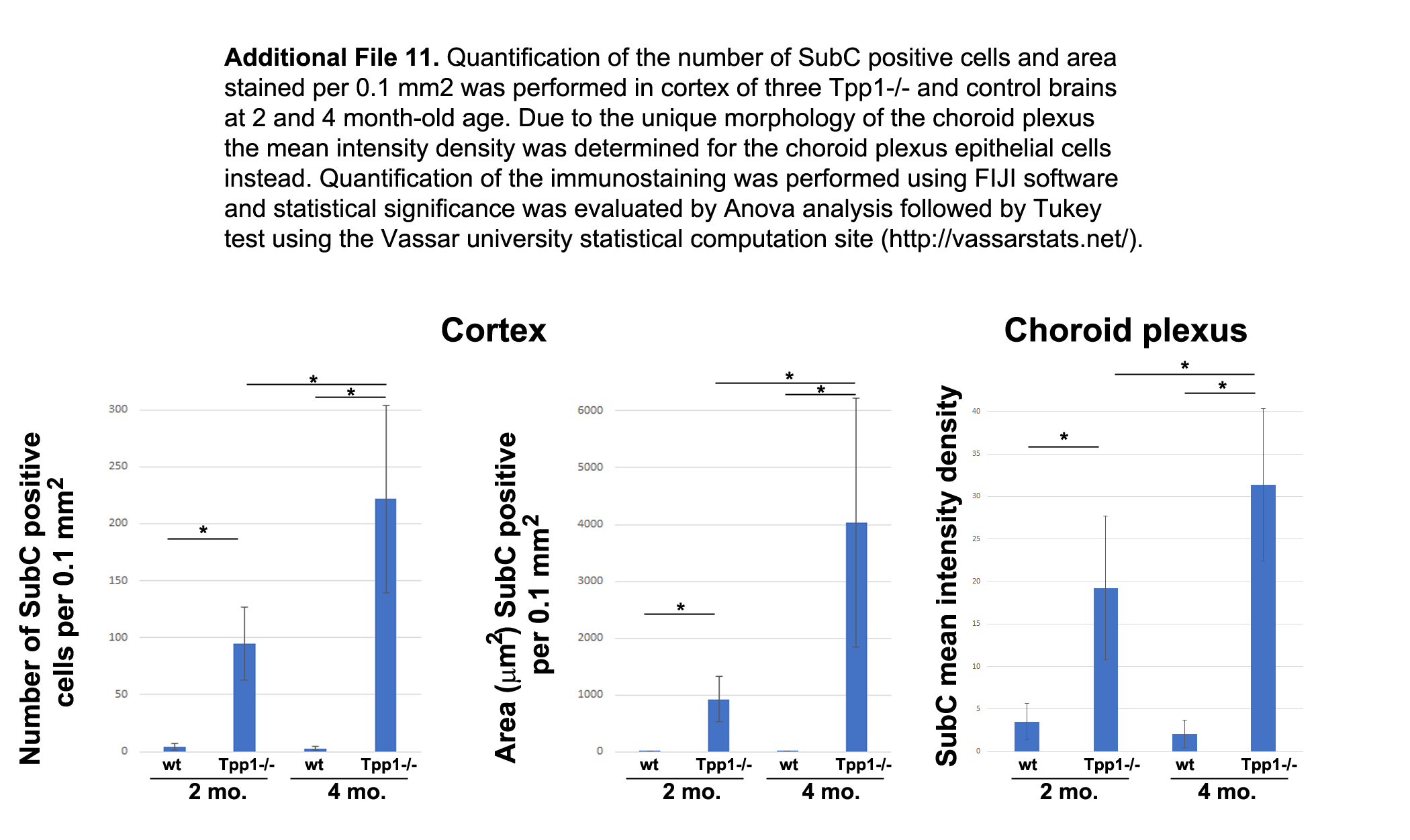

Supplement: Supplementary file 11 — Additional file 11. Quantification of the number of SubC positive cells and area stained per 0.1 mm2 was performed in cortex of three Tpp1−/− and control brains at 2 and 4 month-old age. [file 12974_2021_2302_MOESM11_ESM.tif]
