# Supplementary material for: Brain transcriptome analysis of a CLN2 mouse model as a function of disease progression
Source: J Neuroinflammation. 2021 Nov 8;18:262. doi: 10.1186/s12974-021-02302-z (PMC8576919; doi:10.1186/s12974-021-02302-z)
Supplement: Supplementary file 4 — Additional file 4. Relative differential expression of activated astrocyte signature genes (FPKM in Tpp1−/− relative to control) at different ages. [file 12974_2021_2302_MOESM4_ESM.pdf]

Additional File 4. Relative differential expression of activated astrocyte signature genes (FPKM in *Tpp1*$^{-/-}$ relative to control) at different ages.

		F/M					Cb			
	Gene	1mo	2mo	3mo	4 mo		1mo	2mo	3mo	4mo
PAN-Reactive	Lcn2	0.83	0.43	0.68	1.46		0.72	0.27	0.99	5.75
	Steap4	1.21	1.32	1.04	1.52		1.38	1.13	2.60	1.60
	S1pr3	1.02	0.96	0.94	1.10		0.89	0.82	1.61	3.59
	Timp1	ND	ND	2.82	3.28		ND	ND	4.98	9.30
	Cxcl10	ND	2.59	12.18	17.47		ND	0.93	6.38	7.41
	Cd44	1.09	0.95	1.14	1.19		1.06	1.16	1.66	2.31
	Osmr	1.16	1.25	1.53	1.56		0.93	1.13	1.71	2.65
	Serpina3n	1.07	0.89	2.10	2.55		1.37	0.53	6.00	19.44
	Aspg	1.08	1.05	1.89	1.79		0.99	1.01	1.77	3.37
	GFAP	1.14	1.25	2.64	3.31		1.28	1.07	3.39	7.29
A1-reactive	C3	1.14	0.85	1.85	2.41		1.35	0.95	3.85	11.16
	H2-T23	1.10	0.87	1.14	1.36		1.13	0.83	1.28	1.59
	H2-D1	1.02	1.00	1.37	2.07		1.15	0.99	1.12	1.16
	Ggta1	1.01	0.96	1.50	1.17		1.14	0.98	1.10	1.98
	Iigp1	0.86	1.12	1.76	2.45		0.60	1.08	1.24	2.66
	Gbp2	1.11	1.16	1.71	2.06		0.95	1.07	1.37	2.40
	Fbln5	1.04	1.39	0.95	1.08		1.16	1.16	0.95	1.89
	Fkbp5	1.20	1.01	1.30	0.92		1.31	0.93	1.66	1.41
	Psmb8	1.07	0.88	1.98	2.21		1.28	0.71	1.19	2.04
A2-reactive	Tgm1	ND	ND	ND	10.26		ND	ND	ND	30.24
	Ptx3	ND	ND	ND	0.80		ND	ND	ND	3.18
	S100a10	1.09	0.86	1.09	1.04		1.14	1.01	1.32	1.50
	Cd109	0.86	1.49	1.02	1.39		1.07	0.89	1.42	3.12
	Ptgs2	1.10	0.63	0.89	0.75		0.71	0.71	0.88	2.17
	Cd14	0.96	1.08	1.56	1.72		0.99	1.21	2.91	3.79

ND: Not detected
